# Combination effects of environmental tobacco smoke exposure and nutrients supplement during pregnancy on obesity in Chinese preschool children

**DOI:** 10.3389/fped.2024.1423556

**Published:** 2024-09-13

**Authors:** Wen-Xuan Zhang, Esben Strodl, Wei-Kang Yang, Xiao-Na Yin, Guo-Min Wen, Deng-Li Sun, Dan-Xia Xian, Ya-Fen Zhao, Wei-Qing Chen

**Affiliations:** ^1^ Department of Epidemiology, School of Public Health, Sun Yat-sen University, Guangzhou, China; ^2^ School of Psychology and Counselling, Queensland University of Technology, Brisbane, QLD, Australia; ^3^ Women’s and Children’s Hospital of Longhua District of Shenzhen, Shenzhen, China; ^4^ Department of Epidemiology, School of Public Health (Shenzhen), Sun Yat-sen University, Shenzhen, China

**Keywords:** childhood obesity, environmental tobacco smoke (ETS), nutrients supplement, preschool children, combination effects

## Abstract

**Objective:**

This study aimed to explore the combination effects of prenatal exposure to environment tobacco smoke (ETS) and nutrients supplement during pregnancy on childhood obesity in preschoolers.

**Methods:**

A cross-sectional study was conducted with 58,814 child-mother dyads from 235 kindergartens in Longhua District of Shenzhen, China in 2021. A self-administered structured questionnaire was completed by mothers to collect socio-demographic characteristics, prenatal ETS exposure, and nutrients supplement in pregnancy, and preschoolers' heights and weights were measured at the same time. After controlling for potential confounding variables, logistic regression models and cross-analyses were used to examine the independent and combination effects of maternal prenatal ETS exposure and nutrients supplementation during pregnancy on obesity in preschool children.

**Results:**

The results of our study showed that prenatal ETS exposure increased the risk of childhood obesity (AOR = 1.22, 95% CI = 1.11–1.34) in preschoolers. In addition, risk of childhood obesity was significantly higher when mothers didn't take supplements of multivitamins (AOR = 1.12, 95% CI = 1.05–1.20), folic acid (AOR = 1.23, 95% CI = 1.10–1.37) and iron (AOR = 1.11, 95% CI = 1.04–1.19) during pregnancy. The cross-over analysis showed that the combination of prenatal ETS exposure with mothers taking no multivitamins (AOR = 1.40, 95% CI = 1.21–1.62), no folic acid (AOR = 1.55, 95% CI = 1.12–2.14) and no iron (AOR = 1.38, 95% CI = 1.19–1.59) during pregnancy also increased the risk of obesity among Chinese preschoolers. We also discovered additive interactive effects between prenatal ETS exposure and no maternal multivitamin, folic acid and iron supplementation in pregnancy on the risk of obesity in preschoolers.

**Conclusion:**

The combination of prenatal exposure to ETS with no supplementation of these nutrients might jointly increase the risk of childhood obesity. Public health interventions are needed to reduce prenatal exposure to ETS and to encourage mothers to take appropriate multivitamin, folic acid and iron supplements during pregnancy.

## 1 Introduction

Childhood obesity has become a major public health problem worldwide, with the global childhood obesity rate increasing year by year. According to a survey of 128.9 million people from 1975 to 2016 (1), the global obesity rate for the age range of 5–19 years, increased from 0.7% in 1975 to 5.6% in 2016 for girls, and from 0.9% in 1975 to 7.8% in 2016 for boys, with estimates of 50 million girls and 74 million boys being obese worldwide by 2016. In line with the global obesity epidemic, the obesity rate of children in China has also shown an increasing trend. For example, according to the Health and Nutrition Survey (2), the childhood obesity rate in China increased from 5.3% in 1991 to 16.2% in 2015 in children and adolescents aged 6–17 years. These increasing prevalence rates are concerning given that childhood obesity is likely to persist into adulthood (3, 4), and is an important risk factor for the development of chronic diseases in adulthood such as type 2 diabetes, cardiovascular disease, chronic kidney disease and cancer (5, 6), many mental health problems and premature death (7).

Childhood obesity is considered a multi-factorial metabolic disease (8). From the perspective of Developmental Origins of Health and Disease (DOHaD) hypothesis (9, 10), the occurrence of childhood obesity is influenced by exposure to a range of risk and protective factors during early life (i.e., pre-pregnancy, pregnancy and infancy). Early life may be a critical period for the development of excess weight children due to it being a time of strong body cell division and differentiation, as well as a key period when the formation of tissues and organs are particularly sensitive to external stimuli (11). Exposure to a variety of risk factors during pregnancy may therefore lead to metabolic “programming” that can alter postpartum susceptibility to obesity. This metabolic programming persists into adulthood and plays an important role in the development of chronic diseases in adulthood (12, 13). According to the DOHaD hypothesis, if the fetus is stunted due to intrauterine malnutrition, it will attempt to overcome these limitations by adopting a thrifty energy phenotype (14, 15) which can lead to the occurrence of low birth weight (LBW). When then encountering a nutrient-rich postpartum environment, this mismatch between the state of the postpartum and intrauterine nutritional environments may accelerate the growth of infants and young children and result in the childhood obesity (16).

The most direct cause of intrauterine malnutrition is maternal malnutrition during pregnancy. Our previous research found that inadequate folic acid supplementation during pregnancy was associated with an increased risk of obesity in preschoolers born with SGA, and that this relationship was altered by prenatal multivitamin and iron supplementation (17). However, intrauterine malnutrition is not only related to the mother’s nutrient supplementation, but also to the normal function of the placenta (18). The placenta and umbilical cord are the most important organs connecting the mother and the fetus: being responsible for transporting nutrients and oxygen, and timely discharging the fetal metabolic waste (19). If the placenta is damaged or underdeveloped, it may not be able to deliver enough nutrients and oxygen from the mother to the fetus, eventually leading to intrauterine growth restriction or LBW (20). Our previous studies have shown that prenatal exposure to cooking oil fumes can impair placental development or alter the structure and function of the placenta (21). Several studies have also shown that prenatal exposure to air pollution can lead to an increase in placental vascular resistance (22, 23). These results suggest that exposure to air pollution may affect the function of the placenta, thereby creating a malnourished environment for the fetus.

**Figure 1:**
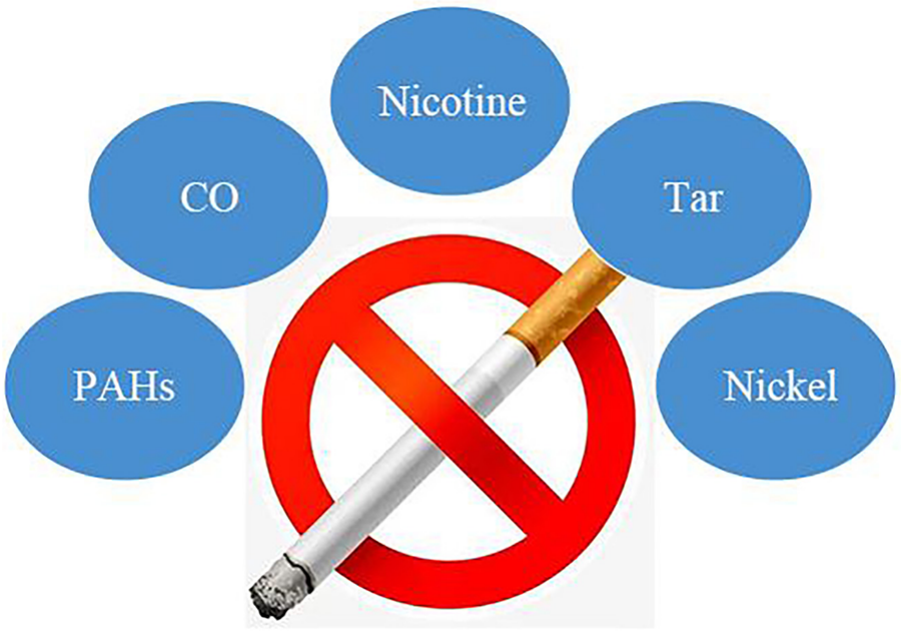
The main harmful components of environmental tobacco smoke. CO, carbon monoxide; PAHs, polycyclic aromatic hydrocarbons.

Environmental tobacco smoke (ETS) is one of the main sources of indoor air pollution (24). ETS contains more than 7,000 chemicals (25), with the main harmful components including nicotine, tar, carbon monoxide (CO), polycyclic aromatic hydrocarbons (PAHs), nitrogen oxides, nickel, cadmium (26, 27) (see Figure 1). Currently, the rate of maternal exposure to ETS during pregnancy is high in many countries. For example, a retrospective cohort study by Crane et al. found that the rate of prenatal exposure to ETS was 11.1% (28), and two studies from Saudi Arabia and Spain reported that the proportion of women exposed to ETS during pregnancy was 31.7 and 55.5%, respectively (29, 30). In China, a survey of 15 provincial and municipal health care institutions across the country by Wang et al. found that 20.2% of women were exposed to ETS during pregnancy (31).

Our previous research showed that prenatal exposure to mosquito coil smoke (MCS) was associated with an increased risk of childhood obesity (32). Moreover, a meta-analysis reported that children with mothers smoking during pregnancy had a 50% increased risk of being overweight compared to children with non-smoking mothers in pregnancy (33). Similarly, a study by Durmus et al. reported that children whose mothers smoked during pregnancy had an increased risk of obesity at age 4 (34). However, the effect of prenatal ETS exposure on obesity in preschool children has not been studied. In addition, given the emerging evidence that maternal prenatal consumption of nutritional supplements may be protective for LBW, we believe it is plausible that this may also be protective for obesity in young children. Moreover, the combination effects of prenatal ETS exposure and nutrients supplement on childhood obesity is unclear. This study therefore aimed to explore the relationship between prenatal ETS exposure, and maternal nutritional supplementation with childhood obesity. We hypothesized that (1) maternal prenatal ETS exposure would be a risk factor; (2) maternal prenatal nutritional supplementation would be a protective factor for childhood obesity; and (3) the combination of prenatal ETS exposure and a lack of nutrient supplementation will show an interactive effect upon the risk of childhood obesity.

## 2 Materials and methods

### 2.1 Setting and subjects

**Figure 2:**
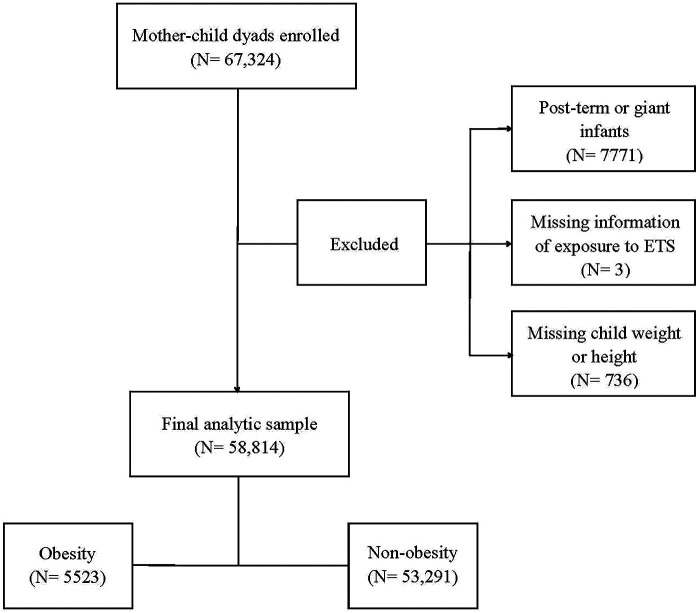
Flow chart of the analytic sample selection process.

A population-based survey was conducted from October to December 2021 in 235 kindergartens in the Longhua District of Shenzhen, China, with a total of 67,324 child-mother dyads recruited. Within this sample, 7771 preschool children born post-term (gestational age >42 weeks) or as giant infants (birth weight >4,000 g) were not included in this study. Three child–mother pairs were excluded due to missing information on prenatal exposure to environmental tobacco smoke, and 736 dyads were excluded due to missing data on the weight or height of the child. This resulted in a total of 58,814 child–mother pairs being included in the final analysis (Figure 2).

The study was approved by the Ethic Committee of the School of Public Health of Sun Yat-sen University. A written informed consent was obtained from the mothers of all the children involved in the study, in accordance with the Declaration of Helsinki.

### 2.2 Data collection

The following process was utilized to develop the self-administered structured questionnaires used in this study. First, we set up a research group and clarified the purpose and object of this study: that was, the effects of exposure to various environmental factors in early life on the obesity in Chinese preschool children. Second, we read a large number of domestic and foreign literature, and referred to the formative questionnaires used in past studies. In addition, from this literature review we identified various environmental factors that a child may be exposed to at various early stages of life. From these activities, we developed the initial draft of the content and structure of the questionnaire. Third, we liaised with experts from the Women’s and Children’s Hospital of Longhua District of Shenzhen on how to design the questionnaire’s questions and the answers, and arrange them in a logical and chronological order. Fourth, as a result of these discussions, we adjusted the layout of the draft questionnaire, including the adjustment of the language, the control of the time of filling in the questionnaire, and the arrangement of the cover instructions and the confidentiality explanation. Fifth, before the formal survey, we conducted a pilot survey and reliability and validity tests. We had made timely changes to the problems identified during the pilot survey, including improving the wording of questions that were poorly understood, the unreasonable option settings, and adding more comprehensive wording to improve the readers’ understanding of some variables, such as the frequency and amount of exposure. Finally, after repeated discussion and modification by experts in many fields such as epidemiology, child education, child health care and health management, the final questionnaire was determined to be ready for the study.

The enrolled mothers were instructed to complete a self-administered structured questionnaire to collect the following information (1): Social-demographic characteristics of the parents’ age at the childbirth, parents’ education level, household income and parents’ marital status; (2) Child’s birth date and gender; (3) Birth-related information including gestational age and birth weight; (4) Maternal pre-pregnancy weight and height, and weight gain during pregnancy; (5) Maternal household air pollution exposure during pregnancy including environmental tobacco smoke, cooking oil fumes, mosquito coil smoke and incense burning smoke.

### 2.3 Prenatal ETS exposure measurement

The researchers asked the mothers the following question to measure their prenatal ETS exposure throughout pregnancy: “Did anyone in the family living with the mother smoke (including e-cigarettes) during pregnancy?” (Two answer options: “No” or “Yes”.) If the answer was “yes,” then prenatal ETS exposure during the pregnancy was considered to be present.

The following questions were used to measure the degree of ETS exposure during each trimester of pregnancy: (1) “During your pregnancy in 1–13 weeks (first trimester)/in 14–27 weeks (second trimester)/after 28 weeks (third trimester), how many cigarettes did others smoke per day in your home?” (Five answer options: 1 score for “1–5 cigarettes/per day”, 2 score for “6–10 cigarettes/per day”, 3 score for “11–15 cigarettes/per day”, 4 score for “16–20 cigarettes/per day”, and 5 score for “>20 cigarettes/per day”.); (2) “During your pregnancy in 1–13 weeks (first trimester)/in 14–27 weeks (second trimester)/after 28 weeks (third trimester), how long were you exposed to ETS per day?” (Seven answer options: 1 score for “1–15 min/per day”, 2 score for “16–30 min/per day”, 3 score for “31–45 min/per day”, 4 score for “46–60 min/per day”, 5 score for “61–90 min/per day”, 6 score for “91–120 min/per day”, and 7 score for “>120 min/per day”).

The score of ETS exposure in frequency of cigarettes per day was the sum of each trimester’s cigarette score calculated as in question (1) above. This resulted in a possible score ranging from 0 to 15. For example, if a preschool child’s mother was exposed to 1–5 cigarettes per day during the 1st trimester, 6–10 cigarettes per day during the 2nd trimester and >20 cigarettes per day during the 3rd trimester respectively, then the preschool children’s score would be “1 + 2 + 5 = 8”. Similarly, the score of ETS exposure in average time per day was calculated in the same way based upon the results of question (2) above, producing a possible score ranging from 0 to 21. The ordinal rankings for the score of ETS exposure in number per day were combined to produce 3 ordinal categories (0, 1–4, ≥5) representing never, low, and high frequency of cigarette exposure. Similarly, the ordinal rankings for the score of ETS exposure in time per day were merged to produce 3 ordinal categories (0, 1–5, ≥6) representing never, low, and high average time per day of ETS exposure.

### 2.4 Maternal nutrients supplement measurement

The following question was asked of mothers to measure the prenatal maternal nutrients supplement exposure throughout their pregnancy (17, 35): (1) “Did you take multivitamin supplements during pregnancy?” (2) “Did you take folic acid supplements during pregnancy?” (3) “Did you take iron supplements during pregnancy?” (Two answer options: “No” or “Yes”).

### 2.5 Obesity assessment

Well-trained nurses from Longhua Maternity & Child Healthcare Hospital took standardized measurements of the height and weight of each preschool children. A portable electronic weight scale (fractional value 0.01 kg) was placed on a level ground, and the children were asked to stand in the center of the scale without a hat, barefoot, and wearing close-fitting lightweight clothing. Once the values were stable, the nurses read and recorded the measurements, accurate to 0.1 kg. Height was measured with a column body altimeter (fractional value = 0.1 cm). The column human altimeter was placed vertically against a wall on a horizontal surface. Preschool children were asked to stand barefoot and bareheaded on a pedal, with their heels close together, feet spaced at an angle of 60°, chest raised, abdomen pulled in and eyes looking straight ahead. The nurses slid the slider to the apex of the measured child’s skull and read the measurement at the same eye height as the slider.

Body mass index (BMI) was calculated by dividing weight in kilograms by height in meters squared (kg/m^2^). We used BMI reference values based upon data from two national representative cross-sectional surveys: The National Growth Survey of Children under 7 years in the Nine Cities of China in 2005 and The Physical Fitness and Health Surveillance of Chinese School Students in 2005 (36, 37). The LMS method was used to smooth the BMI, with estimates of L, M, and S parameters, values of percentile and *Z*-score curves which were required were calculated, and then standardized growth charts were generated (38, 39). Adult cut-offs for overweight and obesity at 18 years was used to study the cut-offs for children 2–18 years of age. This study defined obesity as a BMI equal to or greater than the reference values for sex and age (36).

### 2.6 Confounding variables

According to the relevant literature and previous research (17, 20, 40, 41), potential confounding variables included child’s sex, child’s age, parents’ age at the childbirth, maternal prepregnancy BMI, parents’ education level, household income, prenatal exposure to cooking oil fumes, mosquito coil smoke and incense burning smoke during pregnancy, child’s ETS exposure during 0–3 years of age, child’s nutritional status and physical activity frequency of 0–3 years old.

### 2.7 Statistical analysis

The means and standard derivations (SD) were calculated to describe the continuous variables, and frequencies with percentages were calculated to describe the dichotomous or categorical variables. Student’ *t*-tests or chi-square tests were used for comparing between preschool children with and without obesity.

A cross-over analysis was performed to assess the combination effects on obesity between prenatal ETS exposure and nutritional supplementation. The associations between prenatal ETS exposure and nutritional supplementation with obesity were evaluated using unconditional binary logistic regression models, after adjusting for the confounding variables. Odds ratio (OR) and 95% confidence intervals (95% CIs) were presented to show the strength of association. The multiplicative interaction was estimated by the interaction of odds ratio (IOR) in the logistic regression models. If the 95% CIs of the IOR did not span 1, then the multiplicative interaction was considered significant. Moreover, the relative excess risk due to interaction (RERI) and the attributable proportion due to interaction (AP) were calculated. The 95% CIs of RERI and AP spanned 0; the additive interaction was considered non-significant.

*P*-values were two-sided with alpha set at <0.05. The statistical analysis was performed with R statistical software (version 4.1.1).

## 3 Results

### 3.1 Social-demographic characteristics of participants with and without obesity

Table 1 shows the social-demographic characteristics and birth-related information of the participants. Of the included 58,814 preschool children in our study, 5,523 (9.39%) were obese. The mean age was 4.36 (SD = 0.92) years old for preschool children, 28.58 (SD = 4.39) years old for their mothers, and 30.70 (SD = 5.01) years old for their fathers. Maternal pre-pregnancy BMI was 20.68 (SD = 2.86) kg/m^2^. The rates of preterm birth (PTB) and small for gestational age (SGA) were 8.73 and 10.17%, respectively. More than half of mothers (65.24%) and fathers (66.33%) had a college degree or higher, and 84.87% of the families had the income being ≥10,000 CNY per month.

**Table 1:** Baseline characteristics of the participating children with and without obesity.

Demographic characteristics	Total	Obesity		*t*/*χ*^2^	*p*
		Yes	No		
Number of subjects (*N*,%)	58,814	5,523 (9.39)	53,291 (90.61)		
Child’s age [(Mean ± SD) (years)]	4.36 ± 0.92	4.45 ± 0.89	4.35 ± 0.92	−7.46	<0.001
Child’s sex, *N* (%)				207.43	<0.001
Male	30,852	3,406 (61.67)	27,446 (51.50)		
Female	27,962	2,117 (38.33)	25,845 (48.50)		
Preterm birth, *N* (%)				33.10	<0.001
No	53,680	4,926 (89.19)	48,754 (91.49)		
Yes	5,134	597 (10.81)	4,537 (8.51)		
Small for gestational age (*N*,%)				33.40	<0.001
No	52,834	5,085 (92.07)	47,749 (89.60)		
Yes	5,980	438 (7.93)	5,542 (10.40)		
Maternal age [((Mean ± SD) (years)]	28.58 ± 4.39	28.50 ± 4.51	28.59 ± 4.38	1.331	0.183
Paternal age [((Mean ± SD) (years)]	30.70 ± 5.01	30.75 ± 5.13	30.70 ± 5.00	−0.699	0.484
Maternal marital state (*N*,%)				25.78	<0.001
Married	56,995	5,290 (95.78)	51,705 (97.02)		
Others^a^	1,819	233 (4.22)	1,586 (2.98)		
Paternal marital state, *N* (%)				22.66	<0.001
Married	56,996	5,294 (95.85)	51,702 (97.02)		
Others^a^	1,818	229 (4.15)	1,589 (2.98)		
Maternal education level, *N* (%)				38.55	<0.001
Junior high school or lower	8,634	938 (16.98)	7,696 (14.44)		
High school	11,810	1,178 (21.33)	10,632 (19.95)		
College or higher	38,370	3,407 (61.69)	34,963 (65.61)		
Paternal education level, *N* (%)				41.65	<0.001
Junior high school or lower	7,828	868 (15.71)	6,960 (13.06)		
High school	11,973	1,186 (21.47)	10,787 (20.24)		
College or higher	39,013	3,469 (62.82)	35,544 (66.70)		
Household income (CNY/month), *N* (%)				39.02	<0.001
0–9,999	8,893	978 (17.71)	7,915 (14.85)		
10,000–19,999	20,402	1,936 (35.06)	18,469 (34.60)		
20,000–29,999	12,777	1,139 (20.62)	11,638 (21.84)		
30,000–39,999	6,942	590 (10.68)	6,352 (11.92)		
≥40,000	9,797	880 (15.93)	8,917 (16.79)		
Maternal prepregnancy BMI [((Mean ± SD) (kg/m^2^)]	20.68 ± 2.86	21.31 ± 3.10	20.62 ± 2.82	−15.82	<0.001
Single child or not, *N* (%)				106.64	<0.001
No	18,533	1,401 (25.37)	17,132 (32.15)		
Yes	40,281	4,122 (74.63)	36,159 (67.85)		
Environmental tobacco smoke exposure during 0–1 years of age, *N* (%)				3.91	0.048
No	43,515	4,026 (72.90)	39,489 (74.10)		
Yes	15,299	1,497 (27.10)	13,802 (25.90)		
Environmental tobacco smoke exposure during 1–3 years of age, *N* (%)				2.01	0.156
No	44,680	4,152 (75.18)	40,528 (76.05)		
Yes	14,134	1,371 (24.82)	12,763 (23.95)		
Prenatal cooking oil fumes exposure, *N* (%)				15.24	<0.001
No	12,358	1,273 (23.05)	11,085 (20.80)		
Yes	46,456	4,250 (76.95)	42,206 (79.20)		
Cooking fuel type, *N* (%)				20.04	<0.001
No	12,358	1,273 (23.05)	11,085 (20.80)		
Gas and natural gas	40,978	3,765 (68.17)	37,213 (69.83)		
Electricity	4,658	399 (7.22)	4,259 (7.99)		
Coal	471	45 (0.81)	426 (0.80)		
Others	349	41 (0.74)	308 (0.58)		
Prenatal mosquito coil smoke exposure, *N* (%)				25.22	<0.001
No	41,146	3,701 (67.01)	37,445 (70.27)		
Yes	17,668	1,822 (32.99)	15,846 (29.73)		
Prenatal incense burning smoke exposure, *N* (%)				13.55	<0.001
No	53,267	4,926 (89.19)	48,341 (90.71)		
Yes	5,547	597 (10.81)	4,950 (9.29)		
Nutritional status of 0–1 year old, *N* (%)				34.42	<0.001
Poor	812	72 (1.31)	740 (1.39)		
General	14,868	1,218 (22.05)	13,650 (25.61)		
Well	43,134	4,233 (76.64)	38,901 (73.00)		
Nutritional status of 1–3 year old, *N* (%)				133.18	<0.001
Poor	943	65 (1.18)	878 (1.65)		
General	18,935	1,416 (25.64)	17,519 (32.87)		
Well	38,936	4,042 (73.18)	34,894 (65.48)		
Physical activity frequency of 0–1 year old (days/week), *N* (%)				58.48	<0.001
0	311	53 (0.96)	258 (0.48)		
1	2,876	340 (6.16)	2,536 (4.76)		
2–3	11,389	1,151 (20.84)	10,238 (19.21)		
4–6	15,704	1,455 (26.34)	14,249 (26.74)		
7	28,534	2,524 (45.70)	26,010 (48.81)		
Physical activity frequency of 1–3 year old (days/week), *N* (%)				102.91	<0.001
0	259	55 (1.00)	204 (0.38)		
1	3,532	432 (7.82)	3,100 (5.82)		
2–3	14,219	1,443 (26.13)	12,776 (23.97)		
4–6	15,474	1,387 (25.11)	14,087 (26.44)		
7	25,330	2,206 (39.94)	23,124 (43.39)		

^a^ Including unmarried, divorced, remarried, and spouse loss.

Significant differences were observed for the following characteristics between obese and non-obese preschool children: the child’s age, child’s sex, preterm birth, small for gestational age, parents’ marital state, parents’ education level, maternal pre-pregnancy BMI, household income, being a single child, ETS exposure during 0–1 years of age, prenatal exposure to cooking oil fumes, cooking fuel type, mosquito coil smoke and incense burning smoke, child’s nutritional status and physical activity frequency. More details are presented in Table 1.

### 3.2 Associations between prenatal ETS exposure and obesity in preschoolers

Table 2 presents the associations between prenatal ETS exposure and obesity in preschoolers. After adjusting for the potential confounding variables, compared with no prenatal ETS exposure, prenatal ETS exposure significantly increased the risk of obesity in preschoolers (AOR = 1.22, 95% CI = 1.11–1.34). Further, assessing the associations between the prenatal ETS exposure score in number, time per day and obesity in preschoolers, compared with those without the prenatal ETS exposure, participants with a low score (AOR = 1.15, 95% CI = 1.01–1.32) or a high score (AOR = 1.33, 95% CI = 1.08–1.63) on prenatal ETS exposure in number of cigarettes per day had a high risk of obesity. Similarly, participants with a low score (AOR = 1.16, 95% CI = 1.03–1.32) or a high score (AOR = 1.40, 95% CI = 1.08–1.81) of prenatal ETS exposure in average time per day also had a high risk of obesity.

**Table 2:** The associations between prenatal ETS exposure and obesity among Chinese preschool children.

	Total (*N*)	Cases (*n*,%)	COR (95% CI)	AOR (95% CI)^a^
Prenatal ETS exposure				
No	54,698	5,048 (9.23)	1.00	1.00
Yes	4,116	475 (11.54)	1.28 (1.16–1.42)***	1.22 (1.11–1.34)***
The score of prenatal ETS exposure in number per day				
Never	54,698	5,048 (9.23)	1.00	1.00
Low	3,084	342 (11.09)	1.23 (1.09–1.38)**	1.15 (1.01–1.32)*
High	1,032	133 (12.89)	1.46 (1.21–1.75)***	1.33 (1.08–1.63)**
*p*-value			<0.001	<0.001
The score of prenatal ETS exposure in time per day				
Never	54,698	5,048 (9.23)	1.00	1.00
Low	3,505	393 (11.21)	1.24 (1.11–1.39)***	1.16 (1.03–1.32)*
High	611	82 (13.42)	1.52 (1.21–1.93)***	1.40 (1.08–1.81)*
*p*-value			<0.001	<0.001

^a^ Adjusted for child’s sex, child’s age, parents’ age at the childbirth, maternal pre-pregnancy BMI, parents’ education level, household income, ETS exposure during 0–3 years of age, prenatal exposure to cooking oil fumes, mosquito coil smoke and incense burning smoke, nutritional status and physical activity frequency in models.

* *p* < 0.05.

** *p* < 0.01.

*** *p* < 0.001.

Table 3 displays the effects of different trimester of prenatal ETS exposure on obesity in preschoolers. Compared to children without ETS exposure in the whole pregnancy, only those exposed to ETS in all three trimesters of pregnancy (AOR = 1.18, 95% CI = 1.04–1.34) experienced a significantly increased the risk of obesity as preschoolers. In contrast, participants who were only exposed to prenatal ETS in the 1st trimester (AOR = 1.28, 95% CI = 0.82–2.01), in the 3rd trimester (AOR = 1.44, 95% CI = 0.76–2.74), in both 1st and 2nd trimester (AOR = 1.47, 95% CI = 0.88–2.48), or in the both 1st and 3rd trimester (AOR = 1.32, 95% CI = 0.72–2.43) had elevated odds ratios that did not exhibit a statistically significant increase in the risk of obesity due to wide confidence limits presumably due to the relatively low frequency of obesity cases.

**Table 3:** The associations between trimester-specific ETS exposure during pregnancy and obesity among Chinese preschool children.

Trimesters of ETS exposure			Total (*N*)	Cases (*n*,%)	COR (95% CI)	AOR (95% CI)^a^
1st	2nd	3rd				
NO	NO	NO	54,698	5,048 (9.12)	1.00	1.00
YES	NO	NO	183	25 (11.01)	1.34 (0.88–2.04)	1.28 (0.82–2.01)
NO	YES	NO	33	5 (15.31)	1.49 (0.58–3.82)	0.99 (0.30–3.26)
NO	NO	YES	80	14 (9.23)	1.72 (0.98–3.04)	1.44 (0.76–2.74)
YES	YES	NO	119	19 (9.03)	1.57 (0.97–2.55)	1.47 (0.88–2.48)
YES	NO	YES	93	12 (12.77)	1.27 (0.70–2.32)	1.32 (0.72–2.43)
NO	YES	YES	121	12 (12.84)	0.98 (0.54–1.77)	0.90 (0.48–1.68)
YES	YES	YES	3,012	388 (11.44)	1.27 (1.14–1.41)***	1.18 (1.04–1.34)*

^a^ Adjusted for child’s sex, child’s age, parents’ age at the childbirth, maternal pre-pregnancy BMI, parents’ education level, household income, ETS exposure during 0–3 years of age, prenatal exposure to cooking oil fumes, mosquito coil smoke and incense burning smoke, nutritional status and physical activity frequency in models.

* *p* < 0.05.

*** *p* < 0.001.

### 3.3 Associations between prenatal nutrients supplementation and obesity in preschoolers

After adjusting for the potential confounding variables, the results of logistic regressions showed that no prenatal multivitamin (AOR = 1.12, 95% CI = 1.05–1.20), folic acid (AOR = 1.23, 95% CI = 1.10–1.37) or iron (AOR = 1.11, 95% CI = 1.04–1.19) supplementation significantly increased the risk of obesity in preschoolers (see Table 4).

**Table 4:** The associations between prenatal nutrients supplementation and obesity among Chinese preschool children.

Nutrients	Total (*N*)	Cases (*n*,%)	COR (95% CI)	AOR (95% CI)^a^
Multivitamin				
Yes	24,633	2,064 (8.38)	1.00	1.00
No	34,181	3,459 (10.12)	1.23 (1.16–1.30)***	1.12 (1.05–1.20)***
Folic acid				
Yes	54,728	4,996 (9.13)	1.00	1.00
No	4,086	527 (12.90)	1.47 (1.33–1.61)***	1.23 (1.10–1.37)***
Iron				
Yes	23,992	1,944 (8.10)	1.00	1.00
No	34,822	3,579 (10.28)	1.30 (1.20–1.37)***	1.11 (1.04–1.19)**

^a^ Adjusted for child’s sex, child’s age, parents’ age at the childbirth, maternal pre-pregnancy BMI, parents’ education level, household income, ETS exposure during 0–3 years of age, prenatal exposure to cooking oil fumes, mosquito coil smoke and incense burning smoke, nutritional status and physical activity frequency and other two nutrients in models.

** *p* < 0.01.

*** *p* < 0.001.

### 3.4 Combination effects of prenatal ETS and nutrients supplement exposure on obesity

Table 5 shows the results of crossover analysis on combination effects of maternal prenatal exposure to ETS and nutritional supplementation on obesity in preschool children. Compared to preschool children with mothers who took nutritional supplements and had no ETS during pregnancy, participants with prenatal exposure to ETS and had mothers who did not take nutritional supplements during pregnancy had the highest risk of obesity (AOR = 1.40, 95% CI = 1.21–1.62 for ETS exposure and no multivitamin supplementation; AOR = 1.55, 95% CI = 1.12–2.14 for ETS exposure and no folic acid supplementation; and AOR = 1.38, 95% CI = 1.19–1.59 for ETS exposure and no iron supplementation). This was followed by participants with no prenatal nutritional supplementation only (AOR = 1.17, 95% CI = 1.10–1.25 for multivitamins; AOR = 1.29, 95% CI = 1.16–1.45 for folic acid; AOR = 1.15, 95% CI = 1.08–1.23 for iron), and with prenatal ETS exposure and nutritional supplementation (AOR = 1.21, 95% CI = 1.00–1.45 for ETS exposure and multivitamins supplementation; AOR = 1.19, 95% CI = 1.05–1.35 for ETS exposure and folic acid supplementation; and AOR = 1.19, 95% CI = 0.99–1.44 for ETS exposure and iron supplementation). Further interaction analysis obtained significantly additive interactions between ETS exposure and the three nutrient supplements, but no multiple interactions between them.

**Table 5:** The combination effects of prenatal ETS exposure and nutrients supplement on obesity among Chinese preschool children.

Prenatal ETS exposure	Nutrients supplement	Total (*N*)	Cases (*n*,%)	AOR (95% CI)^a^	IOR (95% CI)^a^	RERI (95% CI)^a^	AP (95% CI)^a^
ETS	Multivitamin						
NO	YES	23,089	1,907 (8.26)	1.00			
NO	NO	31,609	3,141 (9.94)	1.17 (1.10–1.25)***			
YES	YES	1,544	157 (10.17)	1.21 (1.00–1.45)			
YES	NO	2,572	318 (12.36)	1.40 (1.21–1.62)***	1.04 (0.87–1.24)	0.08 (0.04–0.12)	0.07 (0.04–0.10)
ETS	Folic acid						
NO	YES	50,976	4,575 (8.97)	1.00			
NO	NO	3,722	473 (12.71)	1.29 (1.16–1.45)***			
YES	YES	3,752	421 (11.22)	1.19 (1.05–1.35)**			
YES	NO	364	54 (14.84)	1.55 (1.12–2.14)**	1.01 (0.76–1.35)	0.06 (0.02–0.10)	0.06 (0.02–0.09)
ETS	Iron						
NO	YES	22,475	1,799 (8.00)	1.00			
NO	NO	32,223	3,249 (10.08)	1.15 (1.08–1.23)***			
YES	YES	1,517	145 (9.56)	1.19 (0.99–1.44)			
YES	NO	2,599	330 (12.70)	1.38 (1.19–1.59)***	1.01 (0.84–1.20)	0.07 (0.03–0.11)	0.07 (0.03–0.10)

^a^ Adjusted for child’s sex, child’s age, parents’ age at the childbirth, maternal pre-pregnancy BMI, parents’ education level, household income, ETS exposure during 0–3 years of age, prenatal exposure to cooking oil fumes, mosquito coil smoke and incense burning smoke, nutritional status and physical activity frequency in models.

** *p* < 0.01.

*** *p* < 0.001.

### 3.5 Sensitivity analysis

We conducted sensitivity analyses after excluding participants with any prenatal exposure to cooking fuel with coal, mosquito coil smoke and incense burning smoke (*n* = 19,862). The results of the sensitivity analyses were similar to the aforementioned findings. More details are presented in Supplementary Tables S1–S4.

## 4 Discussion

To our best knowledge, this is the first study on the combination effects of maternal prenatal ETS exposure and prenatal nutrients supplementation on obesity in Chinese preschool children. Our study found that prenatal exposure to ETS during pregnancy was significantly associated with the increased risk of obesity in preschoolers. The strength of this association increased with the average time of daily exposure to ETS and the average number of lit cigarettes exposed to each day during pregnancy. We propose that the 1st trimester might be the critical period for prenatal ETS exposure increasing the risk of preschoolers’ obesity. In addition, we also found that mothers not taking multivitamin, folic acid and iron supplementation during pregnancy had children with an increased risk of obesity. Furthermore, we discovered additive interactive effects between prenatal ETS exposure and no maternal multivitamin, folic acid and iron supplementation in pregnancy on the risk of obesity in preschoolers.

### 4.1 Associations between prenatal ETS exposure and obesity in preschoolers

Several previous studies have indicated that maternal exposure to tobacco smoke during pregnancy increases the risk of excess weight in children. For example, the CESAR study in the UK showed a positive association between maternal smoking during pregnancy and their child being overweight, with a pooled odds ratio of 1.26 (95% CI = 1.03–1.55) (42). Similarly, a Canadian study found that children born to active smoking mothers had higher BMIs at ages 2 and 3 than children born to non-smoking mothers (43). In addition, two previous studies suggested that prenatal nicotine exposure had a positive effect on the risk of obesity (33, 44). In line with these findings, our study showed that maternal ETS exposure during the pregnancy was associated with the presence of obesity in preschool children. Interestingly, using a cross-over analysis we found that exposure to ETS during the 1st trimester (the 1st trimester only, both the 1st and 2nd trimester, both the 1st and 3rd trimester marginal significantly and the all three trimesters of pregnancy significantly) increased the risk of preschoolers’ obesity (see Table 3), which suggests that the 1st trimester might be the critical period for the detrimental effect of prenatal ETS exposure on children’s obesity (45). Similarly, several prior studies also found that mothers who smoked in the 1st trimester or throughout pregnancy had a higher risk of obesity in their children (44, 46).

Possible mechanisms for prenatal ETS exposure affecting childhood obesity are as follows. First, prenatal exposure to ETS can alter placenta weight (47), structure (48) and blood vessel function (49). The harmful effects of tobacco smoke are mainly mediated through the release of nicotine and carbon monoxide (CO) (50). Among them, nicotine and CO can all easily cross the placental barrier. Several studies have reported that exposure to nicotine in the mother’s body can result in significantly higher levels of nicotine in the fetal blood than in the maternal blood (51, 52). Nicotine can cause uterine blood vessels to constrict and placental blood vessel resistance to increase by inducing the release of catecholamines (53) and reducing the release of vasodilators such as nitric oxide (NO) (54) from the mother. In addition, CO in tobacco smoke can increase the level of carboxyhemoglobin in the umbilical cord arteries, which aggravates fetal hypoxia (54, 55). This can eventually lead to intrauterine growth restriction (IUGR) and low birth weight (LBW), which was called thrifty energy phenotype (15). According to postnatal catch-up growth theory, the mismatch between IUGR and postnatal excess nutrition may lead to rapid weight gain, which in turn leads to the occurrence of overweight and obesity in early childhood (16). Second, epigenetic changes, especially DNA methylation, are also possible mechanisms by which ETS causes childhood obesity. For example, Novakovic et al. (56) found that hypomethylation of the CpG sites of the *AHRR* gene in the umbilical cord blood of the offspring of smokers persisted until 18 months of age even without exposure to tobacco smoke after birth. *AHRR* gene serves not only to regulate fat metabolism involved in mediating xenobiotic metabolism but is also involved in cell growth and differentiation, further influencing fetal birth weight (57). Our previous study (58) also found that *AHRR* DNA methylation of cord and maternal blood might be associated with LBW. In addition, air pollutants can enter and cross the placenta, so they may have an impact on the health of offspring through changes in placental epigenetic patterns (59–61). Indeed, mothers who lived near major roads, an indicator of traffic-related air pollution, have shown decreased levels of placental *LINE1* (62). Of course, due to the large number of chemicals contained in tobacco smoke, the further research is necessary for fully understanding the mechanisms of prenatal ETS exposure leading to offspring’s obesity in the later life.

### 4.2 Associations between prenatal nutrients supplement and obesity in preschoolers

Maternal nutritional supplementation during pregnancy can also affect the risk of childhood obesity. For example, a Chinese study suggested that pregnant women who take multi-micronutrient (MM) during pregnancy, including iron, folic acid (FA), multivitamin, zinc supplements, and maternal formula, have a lower risk of their offspring being obese at birth and overweight at 3 months of age (63). Similarly, a study in America showed that maternal vitamin B12 (VB_12_) and B6 (VB_6_) concentrations were associated with weight gain in offspring from birth to age 3 (64). In addition, the results of a rat experiment indicated that maternal VB_12_ deficiency led to high triglyceride and cholesterol levels in offspring (65). Moreover, other studies have found that maternal vitamin D (VD) deficiency can also lead to obesity and other obesity-related diseases in offspring later in life (66, 67). Our previous study found that prenatal FA supplementation (OR = 0.72, 95% CI = 0.55–0.93) was associated with a lower risk of obesity in preschoolers (17). Similarly, we also found that no maternal multivitamin, folic acid and iron supplementation during pregnancy significantly increased the risk of obesity in preschool children.

Regarding the potential mechanism, DNA methylation may play a key role in the effect of prenatal nutrients supplement on childhood obesity. In the one-carbon cycle, FA acts as a carbon carrier and VB_12_ as a co-factor of methionine synthase. Both FA and VB_12_ are important regulators of DNA methylation and play an important role in early life development (68). There is growing evidence that FA and VB_12_ changes the methylation status of genes associated with offspring growth (*IGF2*), metabolism (*RXRA*), and appetite control (*LEP*) through consumption of methyl donors, ultimately affecting offspring health (69, 70). For example, *LEP* produces the hormone leptin, which is involved in regulating energy metabolism, making the children eat less, increasing energy release, and inhibiting fat cell synthesis to lose weight (70). *LEP* gene promoter sequence in the cord blood of obese children showed a hypomethylated state (71). At the same time, iron also plays an important role in FA metabolism and one-carbon cycle. When the mothers took FA and iron together during the pregnancy, iron can affect the transcription of FA transporters (72) and regulate the metabolism of one-carbon (73). The mechanism of VD deficiency leading to childhood obesity includes affecting the process of fat formation in offspring, the secretion of adipocytokines, inflammatory response, oxidative stress, etc. (67, 74). An experimental study on rats (75) had found that maternal VD deficiency during pregnancy would promote the proliferation and differentiation of adipocytes in male offspring, and eventually appear obese phenotypes, including an increase in body weight and fat mass. The emergence of this phenotype was likely related to changes in promoter and CpG island methylation levels of several genes, such as the hypermethylation of *Vldlr* gene and the demethylation of *Hif1α* gene.

### 4.3 Combination effects of prenatal ETS exposure and maternal nutrients supplement during pregnancy on childhood obesity

In the present study, we explored the combination effects of prenatal ETS exposure and maternal nutritional supplementation during pregnancy exposure on childhood obesity. We found that maternal nutritional supplementation in pregnancy might reduce the risk of preschoolers’ obesity associated with prenatal ETS exposure. In line with our findings, Wang et al. reported that prenatal mercury exposure was associated with a higher risk of childhood obesity, and adequate intake of FA could reduce this risk (76). Moreover, the cross-over analysis in our study discovered the additive interaction between prenatal ETS exposure and no maternal nutrients supplement during pregnancy on the risk of childhood obesity (see Table 5). These findings suggest that prenatal exposure to ETS and nutritional supplements during pregnancy may have a combined effect on childhood obesity.

Maternal exposure to tobacco smoke during pregnancy had been associated with decreased levels of micronutrients (77, 78), particularly those that play a role in the production of methyl donors. A study in the United States (79) found reductions in one-carbon pathway micronutrients with gestational tobacco smoke exposure, including maternal FA, VB_6_ and VB_12_. We propose the possible following mechanism for the combination effects between prenatal ETS exposure and maternal nutritional supplementation in pregnancy on offspring’s obesity in childhood. In general, higher air pollution exposure was associated with lower methylation of specific CpG sites in GC (61, 80, 81). However, as mentioned earlier, FA as a methyl donor, co-participates in the one-carbon cycle with the assistance of multivitamin (VB_6_ and VB_12_) and iron, and several prior studies (82, 83) found that higher FA intake was often associated with increased levels of methylation. It has been suggested that several components in tobacco smoke, such as organic nitrite, nitrous oxide, cyanate and isocyanate, increase oxidative stress and interact with FA and VB_12_, causing the inactivation of these micronutrients (84). FA and VB_12_ deficiencies also reduce DNA methylation via homocysteine increase, ultimately aggravating the effect of air pollution on DNA methylation (81). This was also verified in a study, which showed that maternal exposure to tobacco smoke during pregnancy was significantly associated with lower FA levels, lower VB_6_ and VB_12_ levels, and increased homocysteine levels (79, 84). Therefore, it may be reasonable to assume that the methylation process, caused by FA supplementation, may counteract the hypomethylation caused by tobacco smoke. In addition, FA could also improve the placental function through anti-inflammatory effects and reduce the risk of IUGR and LBW (85). However, due to the lack of targeted experimental studies, more research is needed to explore the combined mechanisms of ETS and nutrients supplement exposure during pregnancy leading to childhood obesity in the future.

### 4.4 Limitation

The findings of this study need to be interpreted in consideration of the following limitations. First, all participants were recruited from Longhua District of Shenzhen city, which might cause selection bias and limit the generalizability of our findings due to variations in prenatal ETS exposure and nutritional supplementation practices across other areas. Second, the data on ETS exposure in the three trimesters of pregnancy was subjectively recalled by the mother, which might result in memory recall bias and social desirability bias. Third, considering the low rate of active smoking among women of reproductive age in China (86), data on active smoking was not collected. Fourth, unfortunately we did not collect any detailed information about the dose and frequency of maternal nutritional supplementation, or information on the specific vitamins that comprised the multivitamin used by the mothers during pregnancy. This might limit our ability to assess the associations between mothers consuming nutritional supplements during pregnancy and obesity in preschoolers. Fifth, although a range of covariates were included, there were still unmeasured potential confounding variables such as paternal obesity, parental and children diet, household ventilation conditions and ETS exposure in public places during pregnancy which might influence the findings. Sixth, cross-sectional studies limit any conclusions on the causal relationship for the combination effects of maternal prenatal ETS exposure and nutritional supplementation on obesity in preschool children, so the prospective birth cohort studies are needed to determine their causal relationship.

## 5 Conclusions

In conclusion, our study found that maternal prenatal ETS exposure can increased the risk of childhood obesity, while maternal nutritional supplementation during pregnancy can reduce the risk of obesity. Moreover, the combination of prenatal ETS exposure and a lack of maternal nutritional supplementation during pregnancy may jointly affect childhood obesity. These findings support the need for public health interventions to reduce maternal prenatal exposure to ETS and encourage appropriate consumption of multivitamins, folic acid and iron supplements by mothers during pregnancy.

## Data Availability

The datasets presented in this article are not readily available because the datasets generated and/or analyzed during the current study are not publicly available due to privacy protection of the participants, but are available from the corresponding author on reasonable request.
