# Supplementary material for: Combination effects of environmental tobacco smoke exposure and nutrients supplement during pregnancy on obesity in Chinese preschool children
Source: Front Pediatr. 2024 Sep 13;12:1423556. doi: 10.3389/fped.2024.1423556 (PMC11427257; doi:10.3389/fped.2024.1423556)
Supplement: Supplementary file 1 [file Table1.docx]

Supplementary Material

# Supplementary Tables

**Table S1.** The associations prenatal ETS exposure and obesity among Chinese preschool children without prenatal exposure to cooking fuel with coal, mosquito coil smoke and incense burning smoke.

|  | **Total (N)** | **Cases (n,%)** | **COR (95% CI)** | **AOR (95% CI) ^a^** |
| --- | --- | --- | --- | --- |
| *Prenatal ETS exposure* |  |  |  |  |
| No | 36,972 | 3311 (8.96) | 1.00 | 1.00 |
| Yes | 1980 | 217 (10.95) | 1.25(1.08-1.45)^**^ | 1.19(1.01-1.40)^*^ |
| *The score of prenatal ETS exposure in number per day* |  |  |  |  |
| Never | 36,972 | 3311 (8.96) | 1.00 | 1.00 |
| Low | 1509 | 153 (10.13) | 1.15(0.97-1.36) | 1.10(0.91-1.33) |
| High | 471 | 64 (13.59) | 1.60(1.23-2.09)^**^ | 1.49(1.11-2.00)^**^ |
| *P*-value |  |  | <0.001 | <0.001 |
| *The score of prenatal ETS exposure in time per day* |  |  |  |  |
| Never | 36,972 | 3311 (8.96) | 1.00 | 1.00 |
| Low | 1731 | 176 (10.16) | 1.15(0.98-1.35) | 1.12(0.94-1.34) |
| High | 249 | 41 (16.47) | 2.00(1.43-2.81)^***^ | 1.70(1.16-2.49)^**^ |
| *P*-value |  |  | <0.001 | <0.001 |

^a^: Adjusted for child’s sex, child’s age, parents’ age at the childbirth, maternal pre-pregnancy BMI, parents’ education level, household income, ETS exposure during 0-3 years of age, nutritional status and physical activity frequency in models.

*: p<0.05, **: p<0.01, ***: p<0.001

**Table S2.** The associations between trimester-specific ETS exposure during pregnancy and obesity among Chinese preschool children

| **Trimesters of ETS exposure** | | | **Total**  **(N)** | **Cases**  **(n, %)** | **COR**  **(95% CI)** | **AOR**  **(95% CI) ^a^** |
| --- | --- | --- | --- | --- | --- | --- |
| **1st** | **2nd** | **3rd** |  |  |  |  |
| NO | NO | NO | 35,191 | 3114 (8.85) | 1.00 | 1.00 |
| **YES** | NO | NO | 348 | 33 (9.48) | 1.08(0.75-1.55) | 1.15(0.61-2.03) |
| NO | **YES** | NO | 62 | 9 (14.52) | 1.75(0.86-3.55) | 1.30(0.55-3.04) |
| NO | NO | **YES** | 96 | 7 (7.29) | 0.81(0.38-1.75) | 0.78(0.34-1.80) |
| **YES** | **YES** | NO | 178 | 18 (10.11) | 1.16(0.71-1.89) | 1.12(0.67-1.86) |
| **YES** | NO | **YES** | 126 | 14 (11.11) | 1.29(0.74-2.25) | 1.10(0.59-2.06) |
| NO | **YES** | **YES** | 55 | 8 (14.55) | 1.75(0.83-3.71) | 1.95(0.91-4.17) |
| **YES** | **YES** | **YES** | 2896 | 325 (11.22) | 1.30(1.15-1.47)^***^ | 1.30(1.13-1.51)^***^ |

^a^: Adjusted for child’s sex, child’s age, parents’ age at the childbirth, maternal pre-pregnancy BMI, parents’ education level, household income, ETS exposure during 0-3 years of age, nutritional status and physical activity frequency in models.

***: p<0.001

**Table S3.** The associations between prenatal nutrients supplementation and obesity among Chinese preschool children without prenatal exposure to cooking fuel with coal, mosquito coil smoke and incense burning smoke.

| **Nutrients** | **Total (N)** | **Cases (n, %)** | **COR (95% CI)** | **AOR (95% CI) ^a^** |
| --- | --- | --- | --- | --- |
| *Multivitamin* |  |  |  |  |
| Yes | 17,154 | 1387 (8.09) | 1.00 | 1.00 |
| No | 21,798 | 2141 (9.82) | 1.24(1.15-1.33)^***^ | 1.15(1.06-1.24)^**^ |
| *Folic acid* |  |  |  |  |
| Yes | 36,466 | 3220 (8.83) | 1.00 | 1.00 |
| No | 2486 | 308 (12.39) | 1.46(1.29-1.65)^***^ | 1.28(1.12-1.47)^***^ |
| *Iron* |  |  |  |  |
| Yes | 16,265 | 1273 (7.83) | 1.00 | 1.00 |
| No | 22,687 | 2255 (9.94) | 1.30(1.21-1.40)^***^ | 1.15(1.06-1.24)^**^ |

^a^: Adjusted for child’s sex, child’s age, parents’ age at the childbirth, maternal prepregnancy BMI, parents’ education level, household income, ETS exposure during 0-3 years of age, nutritional status and physical activity frequency and other two nutrients in models.

**: p<0.01, ***: p<0.001

**Table S4.** The combination effects of prenatal ETS and nutrients supplement exposure on obesity among Chinese preschool children without prenatal exposure to cooking fuel with coal, mosquito coil smoke and incense burning smoke.

| **Prenatal ETS exposure** | **Nutrients supplement** | **Total**  **(N)** | **Cases**  **(n,%)** | **AOR**  **(95% CI) ^a^** | **IOR**  **(95% CI) ^a^** | **RERI**  **(95% CI) ^a^** | **AP**  **(95% CI) ^a^** |
| --- | --- | --- | --- | --- | --- | --- | --- |
| *ETS* | *Multivitamin* |  |  |  |  |  |  |
| NO | YES | 16,368 | 1311 (8.01) | 1.00 |  |  |  |
| NO | NO | 20,604 | 2000 (9.71) | 1.21(1.12-1.31)^***^ |  |  |  |
| YES | YES | 786 | 76 (9.67) | 1.19(0.92-1.55) |  |  |  |
| YES | NO | 1194 | 141 (11.80) | 1.43(1.16-1.76)^**^ | 1.07(0.78-1.47) | 0.10(0.05-0.14) | 0.08(0.04-0.12) |
| *ETS* | *Folic acid* |  |  |  |  |  |  |
| NO | YES | 34,647 | 3029 (8.74) | 1.00 |  |  |  |
| NO | NO | 2325 | 282 (12.13) | 1.36(1.18-1.56)^***^ |  |  |  |
| YES | YES | 1819 | 191 (10.50) | 1.16(0.97-1.38) |  |  |  |
| YES | NO | 161 | 26 (16.15) | 1.91(1.21-3.02)^**^ | 0.96(0.59-1.58) | 0.07(0.02-0.13) | 0.07(0.02-0.12) |
| *ETS* | *Iron* |  |  |  |  |  |  |
| NO | YES | 15,525 | 1216 (7.83) | 1.00 |  |  |  |
| NO | NO | 21,447 | 2096 (9.77) | 1.18(1.09-1.27)^***^ |  |  |  |
| YES | YES | 740 | 57 (7.70) | 0.95(0.71-1.28) |  |  |  |
| YES | NO | 1240 | 160 (12.90) | 1.54(1.26-1.87)^***^ | 0.69(0.49-0.97) | 0.08(0.04-0.13) | 0.08(0.03-0.12) |

^a^: Adjusted for child’s sex, child’s age, parents’ age at the childbirth, maternal pre-pregnancy BMI, parents’ education level, household income, ETS exposure during 0-3 years of age, nutritional status and physical activity frequency in models.

**: p<0.01,***: p<0.001.
